# Functional characterization of variants of unknown significance in a spinocerebellar ataxia patient using an unsupervised machine learning pipeline

**DOI:** 10.1038/s41439-022-00188-8

**Published:** 2022-04-14

**Authors:** Siddharth Nath, Nicholas S. Caron, Linda May, Oxana B. Gluscencova, Jill Kolesar, Lauren Brady, Brett A. Kaufman, Gabrielle L. Boulianne, Amadeo R. Rodriguez, Mark A. Tarnopolsky, Ray Truant

**Affiliations:** 1 Department of Biochemistry and Biomedical Sciences, Michael G. DeGroote School of Medicine, Faculty of Health Sciences, McMaster University, Hamilton, ON Canada; 2 Department of Ophthalmology and Visual Sciences, McGill University, Montréal, QC Canada; 3 Centre for Molecular Medicine and Therapeutics, BC Children’s Hospital Research Institute, Department of Medical Genetics, University of British Columbia, Vancouver, BC Canada; 4 Division of Neurology, Department of Medicine and Department of Pediatrics, Michael G. DeGroote School of Medicine, Faculty of Health Sciences, McMaster University, Hamilton, ON Canada; 5 Department of Molecular Genetics, University of Toronto, Toronto, ON Canada; 6 Program in Developmental and Stem Cell Biology, Hospital for Sick Children, Peter Gilgan Centre for Research and Learning, Toronto, ON Canada; 7 Vascular Medicine Institute, Department of Medicine, University of Pittsburgh, Pittsburgh, PA USA; 8 Genetic Counselling Program, Hamilton Health Sciences, Hamilton, ON Canada; 9 Division of Ophthalmology, Department of Surgery, Michael G. DeGroote School of Medicine, Faculty of Health Sciences, McMaster University, Hamilton, ON Canada

**Keywords:** Neurodegenerative diseases, Fluorescence imaging

## Abstract

CAG-expanded *ATXN7* has been previously defined in the pathogenesis of spinocerebellar ataxia type 7 (SCA7), a polyglutamine expansion autosomal dominant cerebellar ataxia. Pathology in SCA7 occurs as a result of a CAG triplet repeat expansion in excess of 37 in the first exon of *ATXN7*, which encodes ataxin-7. SCA7 presents clinically with spinocerebellar ataxia and cone-rod dystrophy. Here, we present a novel spinocerebellar ataxia variant occurring in a patient with mutations in both *ATXN7* and *TOP1MT*, which encodes mitochondrial topoisomerase I (top1mt). Using machine-guided, unbiased microscopy image analysis, we demonstrate alterations in ataxin-7 subcellular localization, and through high-fidelity measurements of cellular respiration, bioenergetic defects in association with top1mt mutations. We identify ataxin-7 Q35P and top1mt R111W as deleterious mutations, potentially contributing to disease states. We recapitulate our mutations through *Drosophila* genetic models. Our work provides important insight into the cellular biology of ataxin-7 and top1mt and offers insight into the pathogenesis of spinocerebellar ataxia applicable to multiple subtypes of the illness. Moreover, our study demonstrates an effective pipeline for the characterization of previously unreported genetic variants at the level of cell biology.

## Neurodegenerative disease: Finding new mutations associated with ataxia

Two genetic mutations combine to cause a novel variant of spinocerebellar ataxia (SCA), a rare neurodegenerative disease. SCA affects the cerebellum, a brain region that controls movement, causing progressive coordination problems. Presented with a patient with an atypical form of SCA, Ray Truant at McMaster University in Hamilton, Canada, and co-workers used whole-genome sequencing to identify mutations in two genes: *ATXN7*, involved in cytoskeleton maintenance, and *TOP1MT*, in mitochondria, the cellular powerhouses. Unable to see differences in the affected tissues using routine microscopy, the researchers used computer-guided microscopy image analysis to determine that the altered ATXN7 protein was mis-located in cells. High-fidelity measurements of cell metabolism showed that mitochondria with altered *TOP1MT* produced insufficient energy. These results clarify the genetics of ataxia, and offer new ways to identify the effects of rare mutations.

## Introduction

The autosomal dominant cerebellar ataxias (ADCAs) are a group of inherited neurodegenerative diseases characterized primarily by cerebellar and brainstem degeneration^1^. At present, more than 40 different subtypes exist, with heterogeneous clinicopathological features, and involving varying additional extra-cerebellar and nervous system structures^2^. A large portion of ADCAs are polyglutamine expansion diseases^1^.

Spinocerebellar ataxia type 7 (SCA7) is a polyglutamine expansion ADCA which occurs as a result of a CAG repeat expansion within the first exon of the *ATXN7* gene, located on chromosome 3^3,4^. An expansion of greater than 37 repeats results in the production of a pathogenic polyglutamine tract within the gene’s product, ataxin-7, an 892-residue, 95 kDa protein. Ataxin-7 is an important component of the human SPT3-TAF_II_31-GCN5-L acetyltransferase (STAGA) complex, where it anchors the ubiquitin protease subunit, Usp22, to STAGA, and is critical for both the deubiquitinating (DUB) and histone acetyltransferase (HAT) activity of the complex^5,6^. Within the cytosol, ataxin-7 has been shown to bind to and stabilize microtubules^7^. SCA7 is quite rare, affecting fewer than 1 in 100,000 individuals, and is posited to occur as a result of impaired STAGA activity secondary to a polyglutamine-expanded ataxin-7^8^. Beyond cerebellar atrophy and ataxia, SCA7 is unique from other ADCAs and polyglutamine expansion diseases in that it also involves visual impairment. In addition to causing cerebellar atrophy, polyglutamine-expanded ataxin-7 disrupts transcription of genes regulated by the cone-rod homeobox protein (CRX), resulting in a marked cone-rod dystrophy. This is often the first presenting sign of illness and early stages are readily detectable by electroretinography (ERG)^9^. In addition to impaired STAGA and CRX function, mitochondrial dysfunction is also thought to be a component in the pathogenesis of SCA7. Several clinical reports have characterized mitochondrial abnormalities in liver and skeletal muscle biopsies from SCA7 patients and there is emerging evidence of organelle dysfunction within disease states, although ataxin-7 protein localization has not previously been linked directly to the mitochondria^10–14^.

The mitochondrial topoisomerase I (top1mt), is a 601-residue nuclear-encoded type IB topoisomerase that functions to release tension in circular mitochondrial DNA, permitting normal genetic control and replication^15^. It is crucial for normal cellular function, and top1mt knockout mouse embryonic fibroblasts (MEFs) have been shown to adopt a Warburg phenotype, with impaired oxidative phosphorylation and increased glycolysis and fatty acid oxidation^16^. Moreover, lack of top1mt is associated with increased oxidative stress and accumulation of mitochondrial DNA damage^16^. At the organism level, absence of normal top1mt activity has been shown to result in impaired liver regeneration^17^.

Here, we identify a novel spinocerebellar ataxia variant, occurring in a patient with mutations in both ataxin-7 and top1mt. Unlike SCA7, the mutations observed in ataxin-7 are not a polyglutamine expansion and our patient does not present with cone-rod dystrophy. Through the use of unsupervised, machine-guided microscopy image analysis and high-fidelity measurements of cellular respiration, we find altered ataxin-7 subcellular localization and impaired bioenergetics in disease states. Our work suggests that ataxin-7 may have important interactions with the cellular energetic machinery and provides insight into the phenotype of a new spinocerebellar ataxia variant.

## Materials and methods

### Genotyping and characterization of variants

WES was performed as part of the Finding Of Rare disease Genes (FORGE) Canada Consortium/Care4Rare Canada Consortium research initiative as described previously^18,19^. Follow-up confirmatory testing of mutations in the proband, parents, and sibling was done by Sanger sequencing of *ATXN7* and *TOP1MT*. All participants provided informed consent, signed enrollment forms, and agreed to participate in this study. This study was approved by the Hamilton Integrated Research Ethics Board (#11-427-T). Sequence alignments of variants were created using the National Center for Biotechnology Information’s (NCBI) Constraint-based Multiple Alignment Tool (COBALT)^20^.

### Tissue culture and transfection

Punch skin biopsies were taken from the proband and his parents to establish a primary fibroblast culture. GM02149A wildtype primary human fibroblasts and GM03561A SCA7 primary human fibroblasts were obtained from Coriell Cell Repositories. ND30014 and ND33391 wildtype primary human fibroblasts were obtained from the National Institute of Neurological Disorders and Stroke (NINDS) Human Cell and Data Repository. All cells were cultured in Minimum Essential Medium (MEM; Gibco, Thermo Fisher Scientific) supplemented with 15% fetal bovine serum (FBS; Gibco, Thermo Fisher Scientific) and 1% GlutaMAX (Gibco, Thermo Fisher Scientific) at 37 °C with 5% CO_2_ in an air-jacketed incubator. Cells were cultured in T75 flasks (Sarstedt) and seeded into 35 mm glass-bottom tissue culture dishes for imaging experiments. Transfection of wildtype primary human fibroblasts was done using the Lonza 4D Nucleofector Type-X Electroporator system as described previously^21^.

### Immunofluorescence

Cells were fixed in 4% paraformaldehyde (PFA, Electron Microscopy Sciences) for 30 min at room temperature and subsequently washed three times with phosphate-buffered saline solution (PBS) in 1-min intervals. Next, cells were permeabilized with 0.5% Triton X-100 (BioShop) and 2% FBS (Gibco, Thermo Fisher Scientific) in PBS for 15 min at 4 °C. Following permeabilization, cells were blocked for 2 h in 40-min intervals with 2% FBS (Gibco, Thermo Fisher Scientific) in PBS. Primary anti-ataxin-7 (rabbit polyclonal, Abcam ab11434), anti-top1mt (rabbit polyclonal, Novus Biologicals NBP1-89473), anti-β-tubulin (mouse monoclonal, University of Iowa Hybridoma Bank E7), and TOMM20 (rabbit monoclonal, Abcam ab186734) were diluted in 1% FBS (Gibco, Thermo Fisher Scientific) and 0.02% Tween-20 (BioShop) in PBS at a concentration of 1/50 and incubated overnight for 12 h at 4 °C. Following this, antibody solution was aspirated and cells were blocked for 30 min in three 10-min intervals with 2% FBS (Gibco, Thermo Fisher Scientific) in PBS. Secondary antibodies conjugated to either Alexa488, Alexa594, or Cy5 dye (Life Technologies) were diluted at 1/500 in 1% FBS (Gibco, Thermo Fisher Scientific) and 0.02% Tween-20 (BioShop) in PBS and applied to cells for 1 h at room temperature. Following this incubation, cells were washed with PBS for 40 min in four 10-min intervals and left in PBS for imaging.

### Microscopy and PhenoRipper analysis

Imaging was done using a Nikon Eclipse Ti inverted widefield epifluorescence microscope with either a 60× oil immersion NA1.4 plan apochromat or 40× air NA 0.6 plan fluor objective. A Spectra X LED lamp (Lumencor) was used as the light source, attenuated with ND2 or ND4 filters as necessary. Images were captured by a Hamamatsu Orca-Flash 4.0 CMOS camera. NIS Elements Advanced Research version 4.30 was used for microscope controlling and image acquisition. Qualitative images were generated by obtaining a multichannel Z-stack and performing blind 3D non-iterative deconvolution using algorithms from AutoQuant (Media Cybernetics/Roper Industries, Inc.) within NIS Elements Advanced Research 4.30. Images were captured in a 16-bit non-compressed tagged-image format (TIFF/.tif) and converted to bitmaps (.bmp) or JPEGs (.jpg) using ImageJ64 (National Institutes of Health) prior to analysis. Multichannel images were split into their individual channels using the ImageJ64 (National Institutes of Health) channel splitting function. Images were analyzed using PhenoRipper by selecting three images per cell type at random for thresholding with a block size of 15.

### FRET sensor design and FLIM-FRET

FRET sensors were designed and FLIM-FRET experiments conducted as described previously^22,23^. Briefly, ataxin-7 exon1 was cloned into a modified mCerulean-C1 plasmid, with eYFP cloned into the opposite end of the multiple cloning site (MCS). FLIM was conducted on a Leica TCS SP5 inverted confocal laser-scanning microscope with a 63X glycerol immersion NA1.4 plan apochromat objective. Experiments were conducted in live cells in HEPES buffer and completed in triplicate.

### Seahorse bioenergetic analysis

Oxidative phosphorylation was assessed using the Seahorse XF Cell Mito Stress Test Kit (Agilent). Seahorse XF 24 plates (Agilent) were coated with Matrigel (Corning) to improve adhesion of cell cultures. Each cell line was seeded in triplicate. Oxygen consumption rate was measured sequentially to establish a basal metabolic rate and also to assay for: proton leak, maximal respiration, spare respiratory capacity, ATP production, and non-mitochondrial respiration by treatment with 1 μM of oligomycin, 2 μM carbonyl cyanide-p-trifluoromethoxyphenylhydrazone (FCCP), 1 μM rotenone, and 1 μM antimycin A as outlined by the Seahorse protocol. A bicinchoninic acid assay was performed to normalize all values to protein. Statistical analyses were conducted using Wave software (Agilent) and Prism 6.0 for macOS (GraphPad Software, Inc.).

### Mitochondrial DNA isolation

Skeletal muscle biopsy samples from the patient and three healthy controls were lightly dounce homogenized in 250 mL of Proteinase K buffer. Homogenate was collected and rinsed with an additional 250 mL of Proteinase K buffer to total 500 mL extraction volume. Samples were incubated at 55 °C overnight. Samples were depleted of protein using NaCl on ice, and total DNA precipitated using ethanol and glycogen blue. Samples were resuspended in 150 mL of Tris-ethylenediaminetetraacetic acid (EDTA) buffer (TE; Sigma) containing RNAse (Thermo Fisher Scientific) and 3,4,4′5-tetramethoxystilbene (DMU-212; Sigma). DNA concentration was determined by spectrophotometry (NanoDrop, Thermo Fisher Scientific) after one freeze-thaw cycle.

### Mitochondrial DNA southern blot

DNA samples were digested with BamHI (Fermentas) at 37 °C for 20 min at 1 μg per 20 mL reaction and resolved on a 10 cm pathlength 0.6% agarose gel. Gels were nicked, denatured, neutralized, and transferred on to positively charged Hybond (GE Healthcare) overnight. Next, membrane was pre-hybridized in Church and Gilbert buffer for 2 h then hybridized with randomly labeled human 185 probe overnight. Following this, the membrane was washed three times in 20 min intervals in SSC buffer. The membrane was subsequently exposed to a phosphorimager screen for two days and scanned on a Storm Imaging System (GE Healthcare). The membrane was then re-probed with human mitochondrial DNA probes and similarly processed. The ratio of mitochondrial DNA to nuclear DNA was calculated and normalized to the average of the three control samples.

### Mitochondrial DNA qPCR

Nuclear and mitochondrial DNA levels were determined using human CoxI and GAPDH SYBR assays, respectively. DNA was diluted to 4.6 ng/mL and 2.5 mL was used in a 10 mL reaction (SYBR Green PCR Master Mix, Thermo Fisher Scientific). The assay was performed in triplicate and averaged per sample. Mitochondrial DNA levels were determined relative to the average of the three control samples using the 2(-Delta Delta C(T)) method^24^.

### 2D-IMAGE

Two micrograms of DNA were loaded on a 25 cm 0.4% SeaKem Gold (Lonza) agarose gel and run in 0.5X Tris-borate-EDTA buffer for 16 h at 40 V. Next, each sample lane was excised and soaked in ethidium bromide (Thermo Fisher Scientific) for 20 min. Samples were then placed perpendicular to the original running direction and a 0.4% agarose gel containing ethidium bromide (Thermo Fisher Scientific) was cast around the gel blocks. The gel was resolved for another 16 h at 40 V and then nicked, denatured, and processed per the Southern blot protocol described above^25–27^.

### Mitochondrial stress assay

GM02149A wildtype fibroblasts were cultured in 35 mm glass-bottom tissue culture dishes to approximately 50% confluence. Cells were subsequently treated with either 250 nM, 500 nM, or 1 μM of rotenone for 60 min and fixed in PFA. Immunofluorescence against ataxin-7 was performed as described earlier.

### Drosophila stocks

All fly stocks were maintained on standard cornmeal fly food with a 12/12-h light/dark cycle, at 25 ^o^C and 45–50% relative humidity. The pUASTattB vector, which allows for PhiC31-mediated transgenesis, was used to generate transgenics^28^. Constructs expressing wild-type human ataxin-7 (Q10) and polyglutamine-expanded ataxin-7 (Q169) within a pUASTattB vector were kindly provided by the Juan Botas Lab at Baylor College of Medicine. Human top1mt-GFP was obtained from OriGene Technologies (clone RG208637) and cloned into an empty pUASTattB vector. The Q5 Site-Directed Mutagenesis Kit (New England BioLabs) was used to generate constructs encoding ataxin-7 Q35P, ataxin-7 N556K, top1mt R111Q, and top1mt R111W in the pUASTattB vector backbone using the previously described wildtype constructs. The following stocks (Bloomington *Drosophila* Stock Center) were chosen for microinjection: #24749 for pUASTattB-ATXN7^Q10^, pUASTattB-ATXN7^Q169^, pUASTattB-ATXN7^Q35P^; #24485 for pUASTattB-ATXN7^N556K^; #24482 for pUASTattB-TOP1MT^WT^ and pUASTattB-TOP1MT^R111W^; #24481 for pUASTattB-TOP1MT^R111Q^. Transgenesis was performed by Best Gene, Inc. (Chino Hills, CA). All transgenes were expressed pan-neuronally by crossing to the *elav*^*c155*^*-Gal4* driver (Bloomington Stock Center #458).

### Drosophila longevity and climbing assays

All crosses were established using elav-Gal4 virgin females and males that carried one or two UAS transgenes. All experimental flies were collected within two-to-three days post-eclosion and sorted into separate groups (males or females) of 50 flies per vial under low CO_2_ exposure. All vials were closed by foam plugs and kept horizontally on racks to prevent flies from getting stuck in the fly food. Survivorship was assessed each time flies were transferred to vials with fresh fly food (two-to-three times per week) until all flies died. All experiments were repeated at least three times. Statistica software package, version 13.0 (Statsoft Inc., Tulsa, OK, USA) was used to generate the Kaplan-Meier survivorship curves that were compared using Mantel–Cox log-rank tests for estimation of lifespan values.

Flies assayed for longevity were also used to measure locomotor activity using a countercurrent apparatus at one, three, five, and seven weeks of age. Briefly, each group of 50 flies was placed in an initial vial and left for 60-seconds to adapt followed by a 20-second time interval during which flies were assessed for their ability to climb to the top of the vial^29^. After 20-seconds, the top vial was shifted and the apparatus was tapped to lower flies to the bottom of the next vial where they were given another 20- seconds to climb. This sequence was repeated five times until all flies were given the opportunity to reach the final vial. The number of flies in each of the six vials were counted and a climbing index (CI) was calculated using the following formula CI (the weighted mean) = Σ(*mn*_*m*_)/*N* where *m* is the number of test vial, *n*_*m*_ is the amount of flies in the *m*^th^ vial, *N* is the total amount of flies. CI ranged from one (min) to six (max). For each time point, the data were collected from at least six cohorts per genotype. Two-way analysis of variance with subsequent Tukey HSD post hoc comparisons was applied to evaluate differences among genotypes and age.

All experiments were done in a special room with a 12/12-h light/dark cycle at 25 °C and 60% relative humidity.

## Results

### Clinical presentation of proband and family

**Fig. 1:**
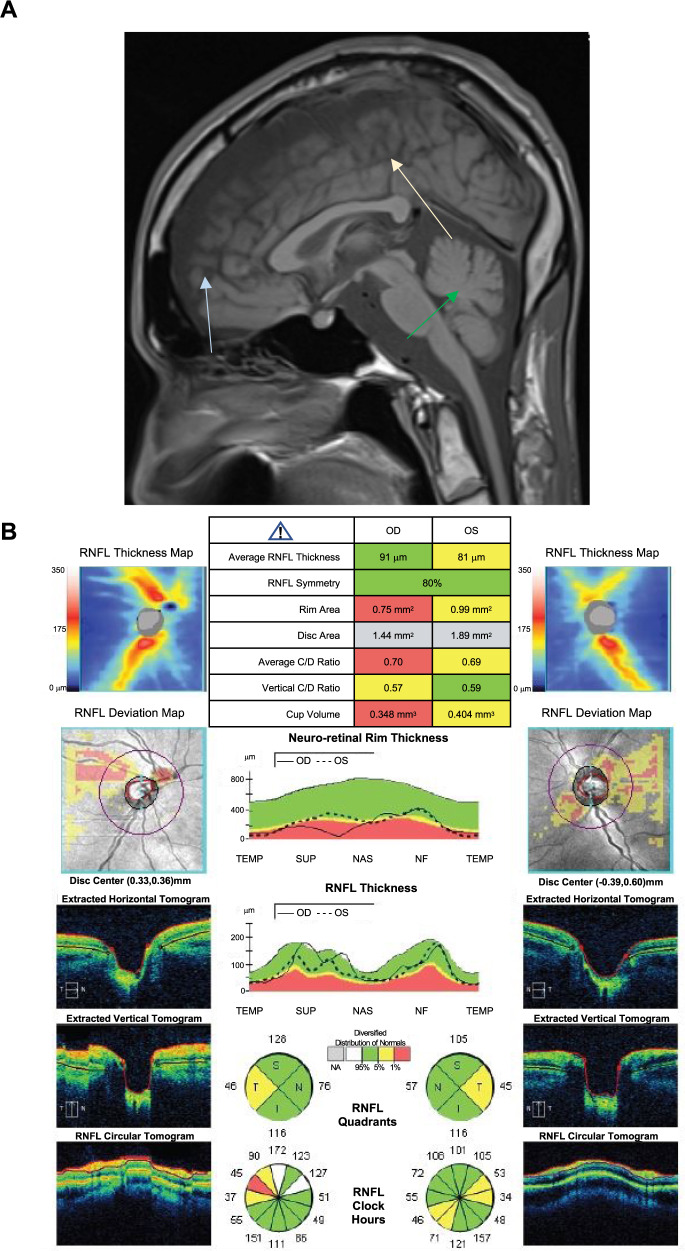
Clinical features of a novel spinocerebellar ataxia variant. **A** T1-weighted sagittal magnetic resonance imaging (MRI) scan showing mild frontal polymicrogyria (beige arrow), frontal cortical volume loss (blue arrow), and cerebellar atrophy (green arrow). **B** Optical coherence tomography (OCT) of the optic nerves of proband. On the left-hand side the analysis of the right eye (oculus dexter, OD) is shown, and on the right-hand side, the analysis of the left eye (oculus sinister, OS) is shown. Thinning of the retinal nerve fiber layer (RNFL) is noted in temporal quadrants in both nerves, corresponding to clinically observed optic disc pallor.

A 32-year-old male was referred to the McMaster University Neuromuscular and Neurometabolic Clinic for examination and work-up of a severe spinocerebellar ataxia of unknown origin. The patient was wheelchair-bound and limited in mobility. He was accompanied by his sister (34-year old), mother (65-year old), and father (67-year old), all of whom were unaffected. The family was identified to be of French-Canadian ancestry. T1-weighted magnetic resonance imaging (MRI) showed mild frontal polymicrogyria, frontal cortical volume loss, and cerebellar atrophy (Fig. 1A). Visual acuity was noted to be counting fingers bilaterally, and the pupils were equal and reactive to light, with no relative afferent pupillary defect. Slit-lamp examination showed a normal anterior segment with no cataracts or corneal deposits in either eye. Fundoscopy revealed temporal pallor of both optic nerves without any evidence of pigmentary retinopathy or vascular attenuation. Gaze-evoked nystagmus was noted bilaterally. Optical coherence tomography (OCT) of the optic nerves showed retinal nerve fiber layer (RNFL) thinning of the temporal quadrant of both nerves (Fig. 1B), corresponding to the clinically observed pallor. ERG was not possible.

### Whole exome sequencing and identification of mutations

**Fig. 2:**
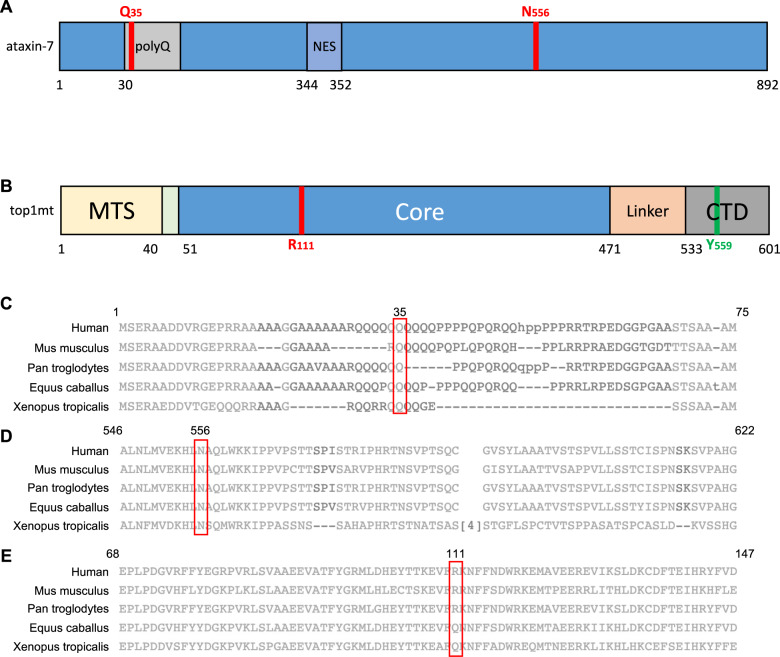
Ataxin-7 and top1mt mutations occur in conserved regions. **A** Schematic of ataxin-7 outlining the polyglutamine (polyQ) tract, the nuclear export signal (NES) and the two affected residues (Q35 and N556). **B** Schematic of top1mt outlining the mitochondrial targeting sequence (MTS), the core domain, the linker, and the c-terminal domain, as well as the catalytic residue (Y559). The affected residue in our proband, R111, is also highlighted. **C** Sequence alignment of human ataxin-7 with additional mammalian and non-mammalian species, demonstrating wide conservation of Q35. **D** Sequence alignment of human ataxin-7 with additional mammalian and non-mammalian species, demonstrating wide conservation of N556. **E** Sequence alignment of human top1mt with additional mammalian and non-mammalian species. Conservation of R111 is seen, although Q is an acceptable variant here, suggesting the R111Q mutation may not be as deleterious. All sequence alignments were generated using the National Center for Biotechnology Information’s Constraint-based Multiple Alignment Tool (COBALT).

Given the patient’s clinical presentation, we elected to perform whole-exome sequencing (WES). Through WES, we identified four variants of unknown significance in two genes, *ATXN7* and *TOP1MT*, on separate alleles. The details of the variants are highlighted in Table 1. The sequence data were compared against major databases of genetic variants, including The SNP Consortium, dbSNP, gnomAD, and ExAC. Nucleotide point mutations resulted in Q35P and N556K mutations within ataxin-7 at the protein level, and R111Q and R111W mutations in top1mt, in both cases on separate alleles. Sanger sequencing of the affected genes within the proband’s family determined the mutations were not *de novo* and established the inheritance pattern (Table 1). Sequence alignments revealed the Q35P and N556K variants within ataxin-7 to occur at highly conserved residues (Fig. 2B, C). The R111Q mutation in top1mt was noted to exist in other mammalian species, while the R111W mutation was noted to be unique to humans, with prior reporting of the mutation in the gnomAD database. Analysis of our mutations by PolyPhen-2 indicated that all but the R111Q mutation were ‘probably damaging’, with R111Q noted as ‘benign’ as it is a substitution found naturally in other mammalian species^30^.

**Table 1:** Distribution of variants.

Gene	Proband	Mother	Father	Sibling
*ATXN7*				
Position: chr3:63898378 Variant: A104C; p.Q35P	+	+	−	−
Position: chr3:63976521 Variant: T1668A; p.N556K	+	−	+	+
*TOP1MT*				
Position: chr8:144411548 Variant: G332A; p.R111Q	+	+	−	+
Position: chr8:144411549 Variant: C331T; p.R111W	+	−	+	−

### Ataxin-7 Q35P has altered subcellular localization and structure

**Fig. 3:**
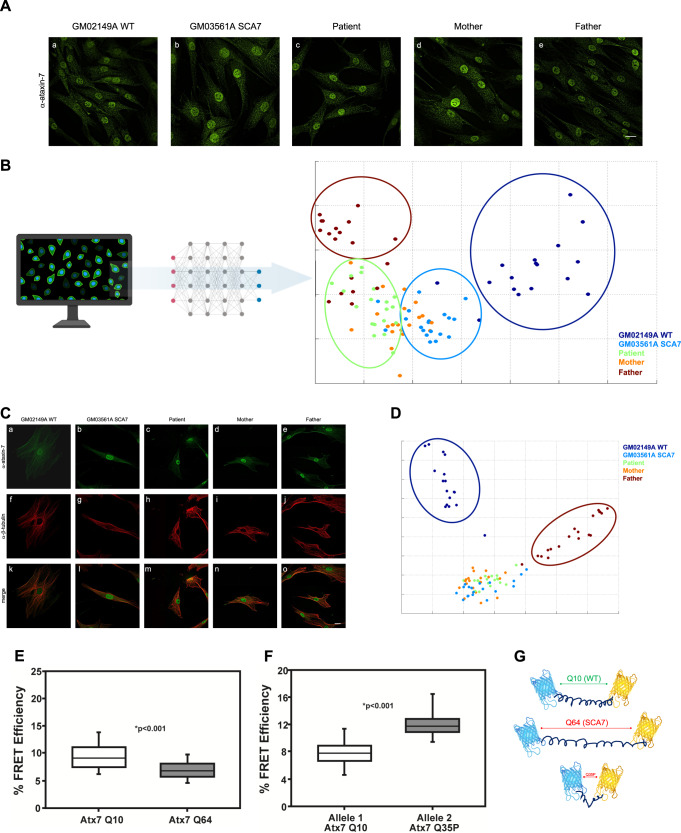
Ataxin-7 Q35P is associated with disease phenotypes. **A** Immunofluorescence of primary human fibroblasts isolated from a healthy individual (a), a SCA7 patient (b), our proband (c), and our proband’s mother (d) and father (e) using antibodies against ataxin-7. No differences in subcellular localization were directly appreciable by eye. Secondary antibodies were conjugated to Alexa488. **B** Schematic outlining PhenoRipper analysis pipeline and multidimensional scaling (MDS) plot of the ataxin-7 immunofluorescence dataset. Cells from our patient’s father, and wildtype cells were noted to sort distinctly within their own groups. Cells from our patient, a SCA7 patient, and our patient’s mother, sorted closer together, indicating similarities between the images. Every point represents one image of 20–25 cells, with 20 images per cell type for *N* ≥ 400 cells per type. **C** Co-immunofluorescence of ataxin-7 and β-tubulin in primary human fibroblasts isolated from a healthy individual (a, f, k), a SCA7 patient (b, g, l), our proband (c, h, m), and our proband’s mother (d, i, n) and father (e, j, o) using antibodies against ataxin-7 and β-tubulin. The first row (a–e) shows the ataxin-7 staining, the second row (f–j) shows the β-tubulin staining, and the third row (k–o) shows the merged channels. Secondary antibodies were Alexa488 for ataxin-7 and Cy5 for β-tubulin. **D** PhenoRipper MDS plot of combined sorting of ataxin-7 and β-tubulin co-stained images. Images were split into individual channels prior to sorting and loaded as two separate sets within PhenoRipper. Images from our patient, SCA7 patient, and our patient’s mother sorted together, while images from our patient’s father and wildtype images sorted distinctly. Every point represents one image of 20–25 cells, with 20 images per cell type for *N* ≥ 400 cells per type. **E**, **F** Box and whisker plots of Förster resonance energy transfer (FRET) efficiency comparing ataxin-7 Q10 (wildtype) to polyglutamine-expanded ataxin-7 (**E**) and Q35P ataxin-7 (**F**). Black lines represent the median value, boxes encompass the 25% and 75% confidence intervals, and whiskers indicate the 5% and 95% confidence intervals. *P* < 0.001, *n* = 100, three replicates. **G** Schematic representations of ataxin-7 FRET sensors. Shown are the wildtype (Q10), polyglutamine-expanded (Q169), and proband (Q35) variants, with their associated distances between donor and acceptor fluorophores. All representative images are post-deconvolution. All scale bars: 10 µm. Figure created with BioRender.com.

To determine the effects of the ataxin-7 mutations on cellular biology, we first performed immunofluorescence against ataxin-7 in skin fibroblasts taken from our patient, his mother and father, a healthy control, and a SCA7 patient. Ataxin-7 is normally localized primarily within the nucleus, with some cytoskeletal staining. No differences in ataxin-7 staining were observed directly by fluorescence microscopy or by review of the images by two separate authors (Fig. 3A). To examine the imaging data in greater depth, we elected to perform unsupervised, unbiased machine-guided analysis using PhenoRipper software^31^. PhenoRipper assesses multiple bitmap texture elements within an image and defines the three most variant textures. These three textures are then plotted in unitless 3D space without predetermined parameters on a multidimensional scaling (MDS) plot of the Principal Component Analysis (PCA) of the three most variant textures in the dataset. Points farthest from one another represent images that are most dissimilar within the dataset while those closest together represent images that are very similar. The PCA plot created from the ataxin-7 immunofluorescence imaging dataset revealed distinct groups of wildtype cells and cells from our patient’s father, while images of cells from the patient, his mother, and the SCA7 patient sorted together, suggesting the Q35P mutation to be associated with a disease phenotype (Fig. 3B).

Given the role of ataxin-7 in stabilizing the cytoskeleton, we next sought to determine whether we could identify any alterations in localization of the protein to microtubules. We performed a co-immunofluorescence of ataxin-7 and β-tubulin across all cell types and again noted no differences in subcellular localization with human observation. PhenoRipper sorting of the dataset revealed a similar pattern as previous, with Q35P ataxin-7 expressing cells observed to sort with SCA7 cells, further suggesting a pathogenic role for this mutation (Fig. 3C, D).

Compelled by these observations and noting the impact of proline as a disruptor of protein secondary structure, we sought next to investigate the impact of the Q35P mutation on ataxin-7 structure. Our group has previously shown, through fluorescence-lifetime imaging microscopy to measure Förster resonance energy transfer (FLIM-FRET), the polyglutamine tract in the Huntington’s disease protein, huntingtin, to function as a flexible hinge allowing for inter- and intra-molecular interactions, with polyglutamine expansion disrupting these^22^. Following that work, we developed FRET sensors with mCerulean blue and enhanced yellow fluorescent protein (eYFP) as donor and acceptor fluorophores, respectively, flanking exon1 of ataxin-7. Our sensors allow for the measurement of intra-molecular FRET, which changes with protein conformation and structure. A baseline FRET efficiency was established using a FRET sensor encoding exon1 of ataxin-7 with 10 glutamines, well below the pathogenic threshold (Fig. 3E). To determine the impact of polyglutamine expansion, as in SCA7, we generated a FRET sensor encoding ataxin-7 exon1 with 64 glutamines. We observed a decrease in FRET efficiency, suggesting that the lengthened polyglutamine tract increased the distance between the donor and acceptor fluorophores (Fig. 3E, G). Finally, we generated a FRET sensor encoding ataxin-7 exon1 with a Q35P mutation. In comparison to the wildtype sensor, we noted a marked increase in intra-molecular FRET with the Q35P sensor (Fig. 3F). Notably, the difference in FRET efficiency between the wildtype and Q35P sensor was much larger than that between the wildtype and polyglutamine expansion sensor (Fig. 3E, F). This suggests there is a large conformational shift with introduction of the proline residue within the polyglutamine tract of ataxin-7 (Fig. 3G).

### Top1mt R111W is associated with bioenergetic deficits

**Fig. 4:**
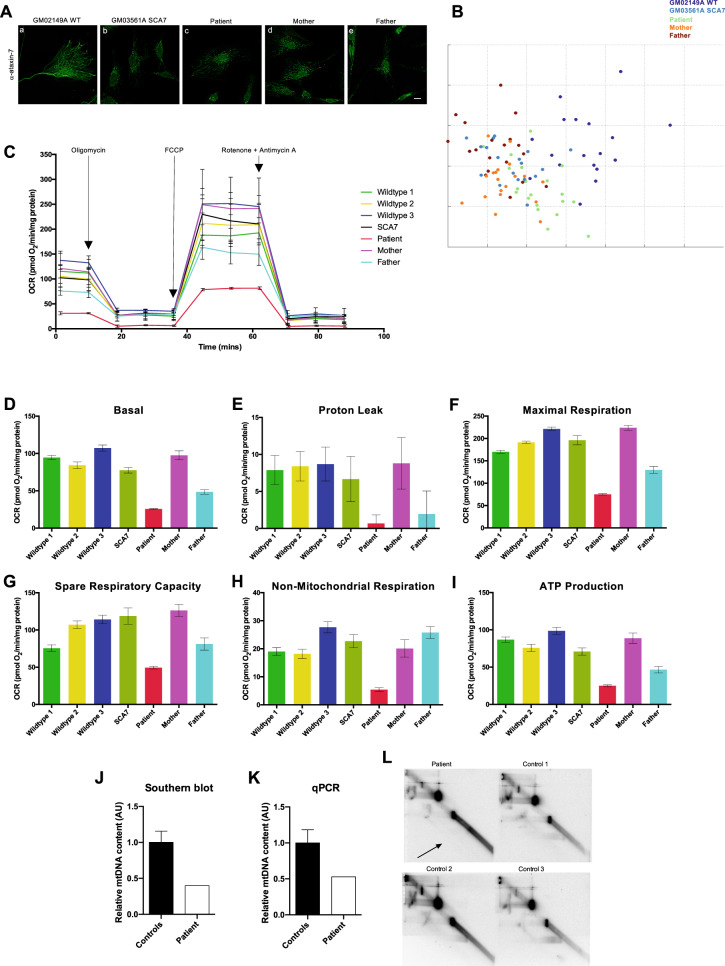
Top1mt R111W is associated with bioenergetic deficits. **A** Immunofluorescence of primary human fibroblasts isolated from a healthy individual (a), a SCA7 patient (b), our proband (c), and our proband’s mother (d) and father (e) using antibodies against top1mt. No differences in subcellular localization were directly appreciable by eye. Secondary antibodies were conjugated to Alexa488. All representative images are post-deconvolution. All scale bars: 10 μm. **B** PhenoRipper multidimensional scaling plot of sorting of top1mt stained images. No discernable groups were observed on sorting. Every point represents one image of 20–25 cells, with 20 images per cell type for *N* ≥ 400 cells per type. **C** Measurement of oxidative phosphorylation across skin fibroblasts obtained from three healthy individuals, our patient, a SCA7 patient, and our patient’s parents using the Seahorse XF 24 Mito Stress Test. Arrows indicate points at which pre-loaded mitochondrial poisons were applied to test different segments of the energetic system. Points are means for triplicates with error bars indicating the standard deviation. Oxygen consumption rate was measured in picomoles of O_2_/min and then normalized to protein per well by bicinchoninic acid assay. Clear defects are noted in cells from our patient, as well as cells from his father, both carrying the top1mt R111W mutation. **D**–**I** Analysis of specific oxidative phosphorylation components including basal respiration (**D**), proton leak (**E**), maximal respiration (**F**), spare respiratory capacity (**G**), non-mitochondrial respiration (**H**), and ATP production (**I**). Notably, for all electron transport chain-dependent components, cells from our patient and his father performed the poorest. All columns represent means with error bars indicating standard deviation. Values were normalized to non-mitochondrial respiration. **J** Southern blot analysis and qPCR analysis (**K**) of relative mitochondrial DNA content from patient skeletal muscle biopsy samples compared to healthy controls, with patient samples having substantially less mitochondrial DNA content. **L** Two-dimensional intact mitochondrial DNA agarose gel electrophoresis (2D-IMAGE) analysis of patient mitochondrial DNA compared to healthy controls. Patient samples were noted to have decreased covalently closed circular DNA levels (arrow) as well as increased linearity.

Given that the Q35P mutation exists also within the proband’s healthy mother, we sought to next determine the impact of the top1mt mutations on cellular biology. We first performed immunofluorescence against top1mt in skin fibroblasts taken from our patient, both parents, a healthy control, and an individual with SCA7. We observed no differences in subcellular localization by eye, nor by PhenoRipper sorting across the different cell types (Fig. 4A, B). Next, in order to characterize mitochondrial morphology, we stained cells with TOMM20, which marks the outer mitochondrial membrane, and included two additional wildtype lines to provide a robust baseline. Once again, we observed no differences in mitochondrial morphology by eye nor by PhenoRipper sorting (Fig. S1A, B).

We next sought to determine whether the top1mt mutations in our proband and his family affect mitochondrial function. Using the Seahorse XF24 System, we profiled the oxidative phosphorylation capacity of patient cells and compared them to cells from his parents, an individual with SCA7, and three healthy controls. Notably, patient cells had the poorest mitochondrial function (Fig. 4C) and this was maintained across all measures within the oxidative phosphorylation analysis (Fig. 4D–I). Cells from our patient’s father (expressing top1mt R111W) were the next lowest in mitochondrial function, suggesting that this mutation may be associated with bioenergetic deficits.

Results of our metabolic profiling were reaffirmed by Southern blot (Fig. 4J) and quantitative PCR (qPCR; Fig. 4K), both of which demonstrated decreased mitochondrial DNA content in patient skeletal muscle biopsy samples when compared to healthy controls. Moreover, we noted alterations in mitochondrial DNA topology in samples obtained from patient biopsy samples when compared to healthy controls via two-dimensional intact mitochondrial DNA agarose gel electrophoresis (2D-IMAGE; Fig. 4L). Most notably, patient cells were observed to contain increased linear mitochondrial DNA and decreased levels of covalently closed circular DNA (Fig. 4L arrow), suggestive of topoisomerase dysfunction, as mitochondrial topoisomerases are responsible for the management of mitochondrial DNA topology in states of replication and at rest.

### Proband mutations in a Drosophila model

**Fig. 5:**
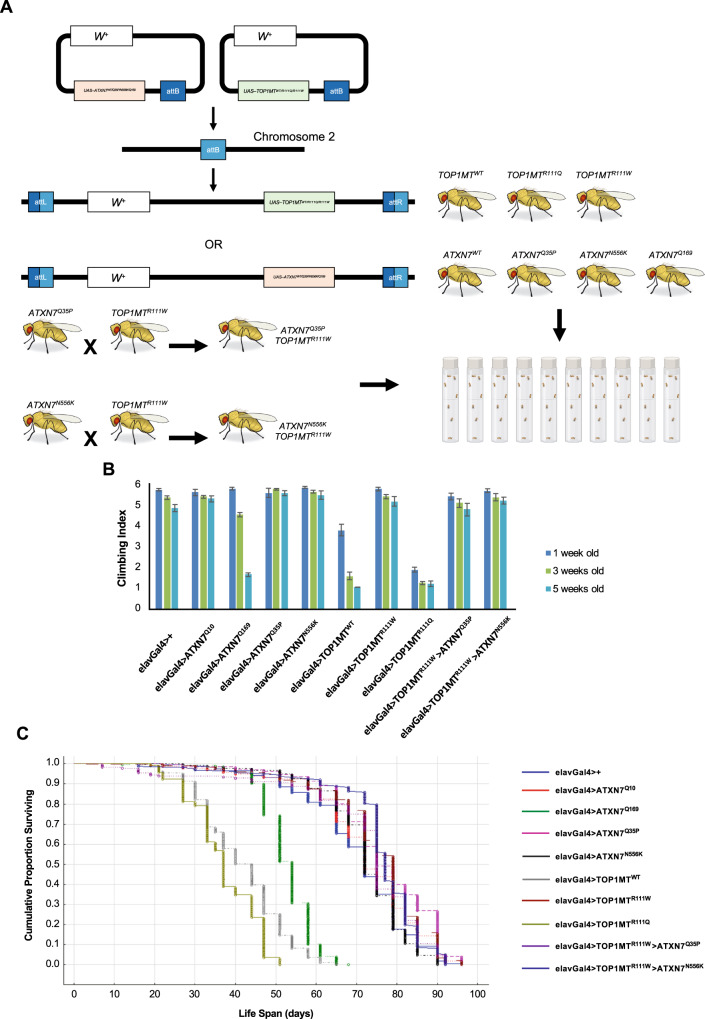
Expression of human ATXN7 (wildtype and mutant variants) and human TOP1MT (wildtype and mutant variants) in the Drosophila central nervous system has variable effects on locomotor activity and lifespan. **A** Schematic representation outlining the development of transgenic flies prior to experimentation. Briefly, we used the pUASTattb vector system which allows for PhiC31-mediated transgenesis. We crossed flies expressing ataxin-7 Q35P and ataxin-7 N556K together with elav-GAL4 with those expressing top1mt R111W to obtain relevant genotypes. **B** Climbing indexes for all control and experimental genotypes described were obtained at one, three, and five weeks of age, with at least six trials per genotype per time point. Experiment was repeated three times. Error bars represent standard error. **C** Kaplan–Meier survival curves for lifespan. Experiment was repeated three times and results combined in the depicted graph. *N* = 200 flies per genotype. Figure created with BioRender.com.

Given that individual mutations associated with pathological phenotypes exist within our proband’s parents separately, we sought to next determine the additive effect of these variants. Specifically, we were interested in the ataxin-7 Q35P mutation and the top1mt R111W mutation, as these exist only within our proband and were observed to individually be associated with aberrant cellular function. We generated *Drosophila* transgenics of all mutations within our proband and family as well as relevant crosses (Fig. 5A). To determine whether *TOP1MT* has any deleterious effects in vivo, we used the GAL4-UAS system to target the expression of wildtype and mutant human *TOP1MT* to the *Drosophila* nervous system using the pan-neuronal driver *elav*^*c155*^*-Gal4*^28^. We first measured locomotor activity using a countercurrent apparatus^29^. As a control, we also included transgenic flies that express *human ATXN7*^Q169^, which has previously been shown to give rise to impairments in locomotor activity in aged flies^32^. We found that expression of *TOP1MT*^*WT*^ and *TOP1MT*^*R111Q*^ in *Drosophila* neurons gave rise to strong locomotor impairments as early as 1 week of age (Climbing Index (CI) = 3.66 ± 0.27 and 1.83 ± 0.12, respectively). We did not observe any defects in *elav-GAL4*controls (CI = 5.52 ± 0.06) or in flies expressing *ATXN7*^Q10^ (CI = 5.37 ± 0.21), *ATXN7*^Q35P^ (CI = 5.37 ± 0.21), *ATXN7*^N556K^ (CI = 5.63 ± 0.04), *TOP1MT*^R111W^ (CI = 5.55 ± 0.08) or *ATXN7*^Q169^ (CI = 5.57 ± 0.06) at this time point (Fig. 5B). Importantly, we found that the locomotor defects observed in *TOP1MT*^*WT*^, *TOP1MT*^*R111Q*^ and *ATXN7*^Q169^ were age-dependent, with the most severe phenotypes observed at 5 weeks of age (Fig. 5B). Of these, *TOP1MT*^*R111Q*^ showed the most severe phenotypes at the earliest time points (CI = 1.21 ± 0.07 at 3 weeks of age). In contrast, we did not observe any significant defects in *elav-GAL4*, *ATXN7*^Q10^, *ATXN7*^N556K^ or *TOP1MT*^R111W^ at either 3 or 5 weeks of age. Moreover, we did not detect any significant locomotor defects in transgenic flies expressing *TOP1MT*^R111W^ with either *ATXN7*^Q35P^ (CI = 4.60 ± 0.29) or *ATXN7*^N556K^ (CI = 5.02 ± 0.16) compared to control *elav*^*c155*^*-Gal4* flies (CI = 4.67 ± 0.16) at either 5 (Fig. 5B) or 7-weeks of age (data not shown). It should be noted that the CI for all genotypes was determined using both males and females except for elavGal4 > *TOP1MT*^R111Q^ flies since the F1 progeny of this cross predominantly gave rise to females with only a few male escapers.

To determine if the observed defects in locomotor activity resulted in reduced longevity, we also measured the lifespan of flies expressing human *ATXN7* or *TOP1MT* transgenes compared to controls (Fig. 5C). The log-rank test indicated highly significant differences in mean lifespan between *elav-GAL4* control flies (72 days) and flies expressing *TOP1MT*^WT^ (44 days) or *TOP1MT*^R111Q^ (37 days). As expected, expression of *ATXN7*^Q169^ also gave rise to flies with a shortened lifespan (54 days) while the lifespan of flies expressing *ATXN7*^Q10^, *ATXN7*^Q35P^, *ATXN7*^N556K^, and *TOP1MT*^R111W^ was similar to controls. As observed for locomotor activity, we did not observe any difference in lifespan in transgenic flies expressing *TOP1MT*^R111W^ with either *ATXN7*^Q35P^ (75 days) or *ATXN7*^N556K^ (77 days).

## Discussion

The clinical, molecular, and functional data presented in this study provide evidence for a previously unreported spinocerebellar ataxia variant, occurring in a patient with concomitant mutations within ataxin-7 and top1mt. Although ataxin-7 has previously been implicated in the pathogenesis of SCA7, we demonstrate that the illness in our proband is in fact a distinct entity. Unlike SCA7, our proband does not suffer from a cone-rod dystrophy. This may be explained by the mechanisms through which SCA7-related visual deficits occur. It has previously been shown that ataxin-7 mediates interactions between the STAGA complex and CRX, which is responsible for the control of multiple genes essential to retinal photoreceptor cell survival^33,34^. Moreover, ataxin-7 itself can directly interact with CRX and polyglutamine-expanded ataxin-7 suppresses CRX transactivation^9^. Although ataxin-7 in our patient has altered structure, it does not contain a polyglutamine expansion, and likely, does not have a similar propensity to aggregate. Thus, it is plausible that CRX function is maintained within our proband, accounting for the lack of pigmentary retinopathy on clinical exam.

Although the ataxin-7 Q35P mutation appears to be quite detrimental, as would be expected with the insertion of a proline residue in the middle of a conserved region, it does not completely explain the disease observed in our proband as his mother also carries the mutation and is completely unaffected. To recapitulate the cause for spinocerebellar ataxia, we must look at mutations in ataxin-7 and top1mt concurrently. Our proband carries not one, but two deleterious mutations, and we propose that these work synergistically to cause disease. To investigate this hypothesis, we created *Drosophila* models, which, interestingly, showed no disease in the crosses carrying our proband’s set of mutations. Moreover, even individually, flies expressing ataxin-7 Q35P and top1mt R111W did not show any locomotor deficits or have shortened lifespans. In fact, in quite the opposite manner, flies expressing top1mt wildtype protein, and top1mt R111Q had severe locomotor deficits and shortened lifespans. To confirm this was not an issue of our *Drosophila* transgenic system, we also created a model with polyglutamine-expanded ataxin-7 (Q169), which exhibited locomotor deficits as expected. Although our transgenic models did not exhibit disease as noted in our proband, they continue to support our hypothesis of the additive effects of the R111W and Q35P mutations. That the R111Q and wildtype top1mt expressing flies showed deficits suggests that there may be protein overload occurring in these organisms. In addition to lack of functional top1mt, overexpression of the protein has also been shown to be associated with deleterious effects on cellular respiration^35^. In fact, top1mt has been posited to be a negative regulator of mitochondrial transcription, unlike other mitochondrial topoisomerases^35^. Thus, it is plausible that expression of R111Q and WT top1mt, both of which are functional, leads to a paradoxical downregulation of mitochondrial transcription within our *Drosophila* transgenics, creating the observed locomotor and lifespan phenotypes. In the case of the R111W expressing transgenics, it is likely that no effect is seen as top1mt R111W is a deleterious variant, with limited function. A protein overload scenario may also serve to explain why transgenics expressing Q35P ataxin-7 do not show locomotor or lifespan phenotypes. It is likely that this variant of ataxin-7 is nonfunctional, given the drastic change in secondary structure, and does not have a dominant-negative effect.

Ataxin-7 and top1mt have not been previously shown to interact, however, there is emerging evidence implicating bioenergetic deficits in SCA7^14^. As ataxin-7 has previously been shown to localize to and stabilize microtubules, where mitochondria are also found, and has both conserved nuclear import and export signals, it is likely that ataxin-7 participates in interactions at the organelle^7,36^. To investigate this further, we treated wild-type skin fibroblasts with rotenone, a potent mitochondrial electron transport chain inhibitor. We observed that ataxin-7 localized to the nucleus in response to mitochondrial stress (Fig. S1C), suggesting a potential for a functional relationship with the bioenergetic machinery. Given that ataxin-7 is an integral component of STAGA, which is responsible for the control of many stress-dependent genes, we hypothesize that ataxin-7 may have additional roles within the cytosol as a sensor of mitochondrial or bioenergetic stress, triggering STAGA-mediated transcription in response.

Beyond delineating the clinicopathological features of a previously unreported spinocerebellar ataxia variant, our study also serves as an example for a novel pipeline for the investigation of uncommon and unreported clinical presentations. Although WES is increasingly becoming the standard for diagnosis and characterization of rare clinical syndromes, to our knowledge, our study is the first to implement WES in conjunction with advanced machine-guided image sorting^18,37^. In cases where clinicians and scientists seek to delineate the impact of novel mutations, machine-guided analyses may assist where conventional cellular and molecular techniques approach their limit.

In summary, we show that concurrent mutations within *ATXN7* and *TOP1MT* are associated with a novel spinocerebellar ataxia variant. Our work offers insight into the cellular biology of both ataxin-7 and top1mt and provides clinicians with an effective pipeline for the characterization of unique variant mutations.

## Supplementary information

Supplemental Figure 1

## Data Availability

The data that support the findings of this study are available from the corresponding author upon reasonable request.
