# Supplementary material for: Functional characterization of variants of unknown significance in a spinocerebellar ataxia patient using an unsupervised machine learning pipeline
Source: Hum Genome Var. 2022 Apr 14;9:10. doi: 10.1038/s41439-022-00188-8 (PMC9010413; doi:10.1038/s41439-022-00188-8)
Supplement: Supplementary file 1 — Supplemental Figure 1 [file 41439_2022_188_MOESM1_ESM.pdf]

Figure S1

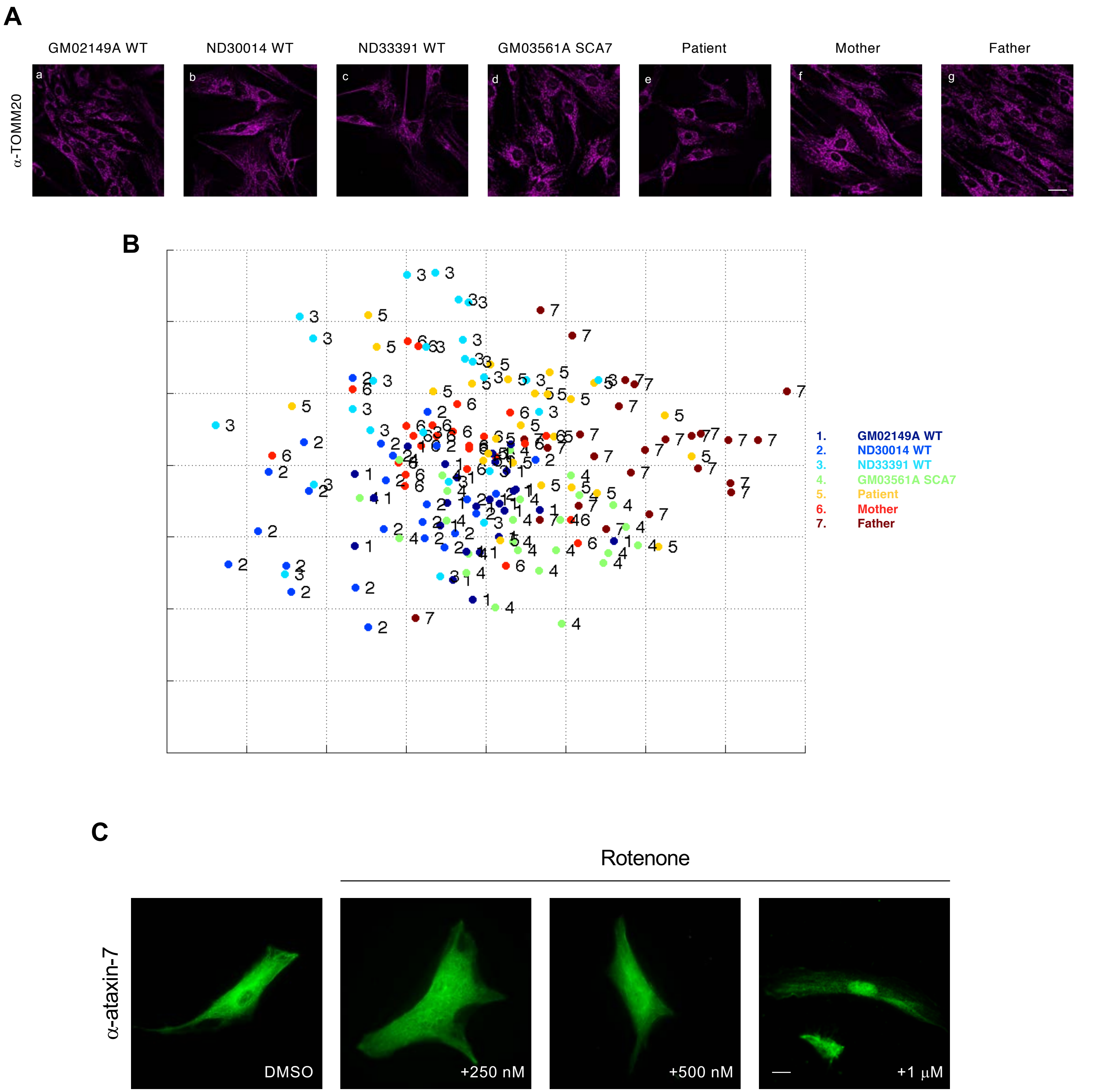

Figure S1: Ataxin-7 translocates to the nucleus in response to mitochondrial stress. (A) Immunofluorescence of primary fibroblasts isolated from healthy individuals (a-c), a SCA7 patient (d), the proband (e), and the proband's mother (f) and father (g) using antibodies against TOMM20, the mitochondrial membrane marker. No differences in staining were obvious. Secondary antibodies were ALEXA594 conjugated. All images were subjected to image deconvolution. Every point represents one image of 20-25 cells, 20 images per cell type for n>400 cells per type. (C) Immunofluorescence of ataxin-7 in wild type GM02149A primary human fibroblasts following treatment with varying concentrations of rotenone for one hour. With 1uM of treatment, increased nuclear localization of ataxin-7 is observed. Representative images were not deconvolved. all scale bars are 10um.
